# Carbon responsibility allocation method based on complex structure carbon emission flow theory

**DOI:** 10.1038/s41598-023-28518-y

**Published:** 2023-01-27

**Authors:** Wenyong Wang, Qunhai Huo, Huawei Deng, Jingyuan Yin, Tongzhen Wei

**Affiliations:** 1 Institute of Electrical Engineering, Chinese Academy of Sciences, Haidian District, Beijing, 100190 China; 2 University of Chinese Academy of Sciences, Shijingshan District, Beijing, 100049 China

**Keywords:** Engineering, Electrical and electronic engineering, Energy science and technology, Renewable energy

## Abstract

A carbon responsibility allocation method based on the complex structure carbon emission flow theory is proposed to address the problem posed by the unclear carbon responsibility allocation of each link in the low-carbon development of electric power. First, the calculation method, distribution characteristics, and mechanism of carbon emission flow were analyzed. The “carbon potential of complex structure” concept was introduced to track “carbon trajectory” and “green trajectory” by harnessing the ability of complex structures to retain two-dimensional information. Subsequently, the carbon responsibility allocation methods for network loss and users’ electricity consumption behavior were developed to realize the accurate carbon responsibility allocation of each system link. Finally, the effectiveness and advancement of the proposed carbon responsibility allocation method were verified using the improved IEEE 6-bus and 30-bus test systems. The application of the proposed complex structure carbon potential in the carbon emission flow theory expands the research dimension of electric power carbon emission for low-carbon development from the “carbon perspective,” provides a novel optimization space for the operation of the distribution network and realizes the carbon emission flow theory, which serves as a bridge from calculation evaluation to optimization decision.

## Introduction

The unclear carbon responsibility allocation of each link in low-carbon electric power development will limit the planning of low-carbon electric power. A reasonable carbon emission reduction plan for the entire link can be provided by clarifying the carbon responsibility allocation of each link during the system operation^1,2^. Current research on low-carbon development mainly focuses on the “electric perspective,” and there is a lack of electric low-carbon studies focusing on the “carbon perspective”^3^ to meet the global carbon goal. In the “carbon perspective,” green becomes the key factor combined with energy security, reliability, and economy.

Recently, extensive research has been performed on developing low-carbon electricity generation from the “carbon perspective” to achieve the “double carbon” goal. The conventional macro statistical^4^ and whole life cycle methods^5^ are based on the conversion of the primary energy consumption. Therefore, they cannot elucidate the space–time transfer mechanism of power carbon emissions and cannot consider power systems’ network and transmission characteristics. Calculating the carbon emission flow of the generating units depends primarily on the path and mode of injection flow of the generating units to the target node. This limits the optimal operation modeling and low-carbon benefit evaluation for low-carbon power generation. Furthermore, it limits the policy formulation of the total carbon emission control and trading systems, green power subsidies, and carbon taxes. Analyzing the allocation of power system carbon responsibility is crucial for stakeholders because it demonstrates the practical applicability of this method.

The carbon emission flow theory is useful in identifying high-carbon elements of a system and presents considerable potential for research on low-carbon power^6,7^. Reference^8^ analyzed a power system’s low-carbon demand response mechanism that employs the dynamic carbon emission factor as the guiding signal. References^9–11^ analyzed the low-carbon planning of multi-energy networks based on carbon emission flow theory. References^12,13^ analyzed the carbon emission reduction planning of an integrated electric-hydrogen system of hydrogen vehicles. References^14–17^ employed carbon emission flow theory and demand response for the optimal scheduling of integrated energy systems. Reference^18^ considered the carbon emission flow theory and demand response to the developed power system low-carbon optimal dispatch, assuming the carbon price as the price signal. Reference^19^ used the method of carbon emission flow to supply power to gas stations and help determine the construction site and capacity. However, these studies did not quantify the carbon emission responsibilities of each link in a power system.

Reference^11^ defined $\lambda$ as a 0 + 1 function to solve the problem of carbon responsibility allocation in lossy networks. During $\lambda = 0$, the power generation side allocates all network losses, and during $\lambda = 1$, all network losses are allocated by the user side. Reference^20^ proposed sharing the carbon responsibility on the power generation and load sides and modeled it as a cost allocation problem based on a cooperative game. Reference^21^ defined an adjustable parameter between 0 and 1. The network loss is then allocated proportionally to the power generation and user sides. However, the carbon emission flow theory still has some drawbacks. This study summarizes the following four points.

1. The carbon reduction contribution of green electricity cannot be quantified, and the carbon reduction effect of green electricity on the user side cannot be reflected. Because the carbon potential of the new energy unit is represented by 0 in the traditional case, it can only reflect the physical carbon emission value of the new energy unit as zero but cannot identify the new energy elements on the user side.
2. It is impossible to compare carbon potential nodes of the same size; therefore, further optimization decisions cannot be made. When the carbon potential of the two nodes is the same, it only reflects that the carbon emissions generated by the two nodes when they consume the same electricity are equal. It is impossible to distinguish the specific proportion of green electricity consumed by the two nodes in this node, which is not conducive to further low-carbon power planning.
3. Lack of a driving force for users to participate in carbon emission reduction. When a node’s load demand increases, other nodes’ carbon potential will increase, and the other nodes will share more carbon emissions, violating the fairness principle of carbon emission sharing.
4. More comprehensive research on carbon emissions from electric power is required. Currently, the distribution and transfer rules of carbon emissions in the power system and the measurement and analysis methods are mainly based on the “electric perspective.” However, a lack of analysis from the perspective of carbon emissions is not conducive to reasonable carbon emission reduction planning for the entire link.

With the rapid development of new energy, some enterprises have increasingly self-built new energy. However, some do not have the power to build new energy because of the factory’s interests. Distinguishing the carbon emission responsibility of different electricity users has become a key problem that needs to be solved. This study analyzes the calculation method of carbon emission flow and its distribution characteristics and mechanism to overcome the problem of the quantitative allocation of carbon emission responsibility in power systems.

The main contributions of this study are as follows:

1. The concept of “complex structure carbon intensity” was proposed because the carbon reduction contribution of new energy units cannot be quantified. The complex structure can retain the characteristics of two-dimensional information and can be used to track the “carbon trajectory” and “green trajectory.”
2. A user-side carbon responsibility allocation method is proposed for the problem of carbon responsibility allocation in the traditional carbon flow theory based on “complex structure carbon potential.” This method can realize the accurate quantitative allocation of user-side carbon emissions and fairness of carbon responsibility allocation.
3. A new method of carbon responsibility allocation for network loss is proposed to solve the problem of carbon responsibility allocation error in a lossy network based on the concept of “complex structure carbon potential.” This method can realize fairness in carbon responsibility allocation for network loss.
4. The improved IEEE 6-bus and 30-bus test system verified the effectiveness of the carbon responsibility allocation method and the method for allocating network loss carbon responsibility based on the “complex structure carbon intensity” theory.

Applying the proposed complex structure carbon intensity in the carbon emission flow theory expands the research dimensions of power carbon emissions for low-carbon development from the “carbon perspective.” This presents a new optimization space for the operation of distribution networks, and the carbon emission flow theory becomes a bridge between calculation evaluations and optimization decisions. The remainder of this paper is organized as follows. In "Carbon emission flow theory" section presents a theoretical introduction of carbon emission flow. In "Concept of complex structure carbon potential" section defines the concepts of “complex structure carbon intensity” and "carbon emission-green electric amplitude angle". In "Application of complex structure carbon intensity" section presents the proposed user-side carbon responsibility allocation method and the method for allocating network loss carbon responsibility based on the “complex structure of carbon intensity”. In "Example analysis of test system" section presents a verification of the proposed method using the improved IEEE 6-bus and 30-bus test system. In "Practical application of complex structure carbon emission flow theory" section presents a discussion of the practical application of the carbon emission flow of a complex structure. Finally, conclusions are presented in "Conclusion" section.

## Carbon emission flow theory

**Figure 1:**
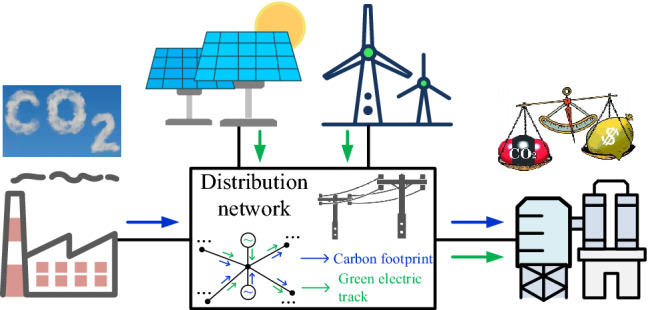
Schematic of carbon trajectory and green electricity trajectory of distribution network.

The carbon emission flow (electricity system carbon emission flow, CEF) is a virtual network flow formed by the carbon emissions attached to the power flow. These are used to characterize the carbon emissions that maintain the power flow of any branch, which is equivalent to adding a carbon emission label to the power flow of each node. The carbon emission starts from the generator set with a nonzero physical carbon emission value, flows in the network with the trend, and is ultimately consumed by the load. Green electricity starts from the generating units with nonzero physical carbon emissions, follows the flow in the network, and is ultimately consumed by the load. Figure 1 presents the distribution network’s carbon and green electricity trajectories.

A system has *N* nodes, of which *K* nodes have a unit injection, *M* nodes have a load, and the network topology is known.

1. The branch power flow distribution matrix is a matrix that describes the power flow distribution of the network. The matrix contains the power flow distribution information and topology information, which is represented by ${\varvec{P}}_{{\text{B}}} = (P_{{{\text{B, }}ij}} )_{{{\text{N}} \times {\text{N}}}}$. When the node, *i* → *j*, has a branch and the flow, *p*, is positive, then $P_{{{\text{B, }}ij}} = p$, in other cases, $P_{{{\text{B, }}ij}} = 0$.
2. The branch network loss distribution matrix is a matrix that describes the distribution of the network loss and is represented by ${\varvec{P}}_{{{\text{Loss}}}} = (P_{{{\text{Loss, }}ij}} )_{{{\text{N}} \times {\text{N}}}}$. When the node, *i* → *j*, has a branch and the forward power flow network loss is *q*, then $P_{{{\text{Loss, }}ij}} = q$, in other cases,$P_{{{\text{Loss, }}ij}} = 0$.
3. The unit injection distribution matrix is a matrix that describes the distribution of the generating units. The matrix contains the location information of the generating units in the network and the power injected into the system, which is represented by ${\varvec{P}}_{{\text{G}}} = (P_{{{\text{G, }}kj}} )_{{{\text{K}} \times {\text{N}}}}$. When the *kth* generating unit is connected to the node, *j*, and the active power flow of the injected node, *j*, is *p*, $P_{{{\text{G, }}kj}} = p$, otherwise, $P_{{{\text{G, }}kj}} = 0$.
4. The load distribution matrix is a matrix that describes the distribution of the power load. It contains the location information of the power load in the network and the amount of active load, expressed by ${\varvec{P}}_{{\text{L}}} = (P_{{{\text{L, }}mj}} )_{{{\text{M}} \times {\text{N}}}}$, when the *mth* power load access is connected to node, *j*, and the active load is *p*, $P_{{{\text{G, }}kj}} = p$, otherwise, $P_{{{\text{G, }}kj}} = 0$.
5. The node active flux matrix describes the power flow injected into the node and is represented by ${\varvec{P}}_{{\text{N}}} = (P_{{{\text{N, }}ij}} )_{{{\text{N}} \times {\text{N}}}}$ with the non-diagonal elements represented as, $p_{{{\text{N, }}ij}} = 0$,$i \ne j$. The node carbon potential is only affected by the injected power flow and remains unaffected by the power flow from the node. For node *j*,
  1 $$P_{{{\text{N, }}jj}} = \sum\limits_{i = 1}^{N} {P_{{{\text{B, }}ij}} } + \sum\limits_{k = 1}^{K} {P_{{{\text{G, }}kj}} }$$
6. The carbon emission intensity matrix of the generator set describes the carbon emission characteristics of the generator set, expressed by ${\varvec{E}}_{{\text{G}}} = (E_{{{\text{G, }}k}} )_{{{\text{K}} \times 1}}$. When the carbon intensity of the *kth* generator set is *e*, then $E_{{{\text{G, }}k}} = e$.
7. The node carbon intensity matrix describes the carbon emission characteristics of the network nodes, represented by ${\varvec{E}}_{{\text{N}}} = (E_{{{\text{N, }}n}} )_{{{\text{N}} \times 1}}$. The physical definition of the node carbon potential is the carbon emission value equivalent to the power generation side caused by the consumption of unit power at the node. For the power plant node, the node carbon potential is equal to the real-time carbon emission intensity of the power plant. When considering the network loss, the node carbon potential, $E_{{{\text{N, }}n}}$, of node, *n*, is expressed as follows:
  2 $$E_{{{\text{N, }}n}} = \frac{{\sum\limits_{i = 1}^{N} {(P_{{{\text{B, }}in}} + \lambda P_{{{\text{Loss, }}in}} )E_{{{\text{N, }}in}} } + \sum\limits_{k = 1}^{K} {P_{{{\text{G, }}kn}} E_{{{\text{G, }}k}} } }}{{\sum\limits_{i = 1}^{N} {P_{{{\text{B, }}in}} } + \sum\limits_{k = 1}^{K} {P_{{{\text{G, }}kn}} } }}$$

where $$E_{{{\text{N, }}in}}$$ represents the branch carbon flow density of branch, *ij*. According to the nature of carbon emission flow, the branch carbon flow density is equal to the carbon potential of the starting node of the branch, i.e., $$E_{{{\text{N, }}in}} = E_{{{\text{N, }}i}}$$;$$\lambda_{{}}^{{}} (\lambda \in [0,1]$$$$)$$ is the network loss allocation coefficient.

Extending the above equation to the full system dimension, the carbon potentials of all the nodes are calculated as follows^6^:

3 $${\varvec{E}}_{{\text{N}}} = [{\varvec{P}}_{{\text{N}}} - ({\varvec{P}}_{{\text{B}}} + \lambda {\varvec{P}}_{{{\text{Loss}}}} )^{{\text{T}}} ]^{ - 1} {\varvec{P}}_{{\text{G}}}^{{\text{T}}} {\varvec{E}}_{{\text{G}}}$$

The load carbon flow rate is expressed as:

4 $${\varvec{R}}_{{\text{L}}} = {\varvec{P}}_{{\text{L}}} {\text{diag}}({\varvec{E}}_{{\text{N}}} )$$

The carbon flow rate of the injection system is expressed as:

5 $${\varvec{R}}_{{\text{G}}} = {\text{diag}}({\varvec{E}}_{{\text{G}}} ){\varvec{P}}_{{\text{G}}} .$$

## Concept of ‘complex structure carbon potential’

Under the traditional calculation method, carbon emissions are apportioned to each unit by the generator set or by the output size, as follows:

6 $$R_{{{\text{G, }}k}} = \frac{{R_{{\sum {\text{G}}}} }}{K}$$

7 $$R_{{{\text{G, }}k}} = \frac{{P_{{{\text{G, }}k}} }}{{{\varvec{\zeta}}_{{\text{K}}} {\varvec{P}}_{{\text{G}}} {\varvec{\zeta}}_{{\text{N}}}^{{\text{T}}} }}R_{{\sum {\text{G}}}}$$

However, the mechanism of generating unit apportionment or output allocation deviates from the principle of fairness according to the carbon responsibility allocation method of the existing carbon emission flow theory, which is not conducive to low-carbon power planning from the ‘carbon perspective’. Conversely, the carbon reduction contribution of new energy units cannot be effectively reflected, and its carbon reduction contribution cannot be quantified. The carbon intensity of new energy units is typically expressed as 0, and $E_{{{\text{G, }}k}} = 0$ only indicates that the physical carbon emission value of new energy units is zero. Additionally, because the new energy unit output level is different, the system contribution to carbon reduction must also be different.

The concept of ‘complex structure carbon intensity’ is proposed in this study based on the above analysis. The complex structure can retain the characteristics of the two-dimensional information, and the carbon intensity is called ‘complex structure carbon intensity’, which can be used as a tool to track the ‘carbon trajectory’ and ‘green trajectory’. The ‘complex structure carbon intensity’ is not a complex carbon intensity. It involves using a complex structure to retain the characteristics of the two-dimensional information, which further improves the carbon emission flow theory. For the convenience of practical application in the carbon emission flow theory, $E_{{{\text{G, }}k}} = < e,g{ > }$ is expressed as $E_{{{\text{G}}, \, k}} = e + gj$, and ${\varvec{E}}_{{\text{G}}}^{{{\text{CN}}}}$ is defined as the node complex structure carbon intensity matrix, where *e* denotes the real part of the carbon potential, *g* denotes the imaginary part of the carbon intensity (the *g* value of the new energy unit is 1), and *j* denotes the complex structure unit. The ‘complex structure carbon intensity’ of the generator set with zero physical carbon emission is represented by (*e* + 0j) tCO_2_/MWh, and the ‘complex structure carbon intensity’ of the generator set with zero physical carbon emission is represented by (0 + j) tCO_2_/MWh. The physical definition of the node's ‘complex structure carbon intensity’ is as follows. Every 1 MWh of electricity consumed by the node produces *e* tCO_2_ emissions, and the proportion of green electricity in each 1 MWh of electricity consumed by the node is g × 100%.

However, the representation of the real part carbon potential and the imaginary part carbon intensity of the node are insufficient to adequately solve all the problems. Furthermore, the angle between the real carbon and imaginary carbon intensities in the complex structure plane is defined as the node ‘carbon emission-green electricity amplitude angle’, $\angle C$, by using the complex structure characteristics.

The complex structure carbon intensity of any node can be obtained using Eq. (3). The complex structure carbon intensity of nodes *p* and *q* can be expressed as:

8 $$\left\{ \begin{gathered} E_{N,p} = e_{p} + {\text{i}}g_{p} \hfill \\ E_{N,q} = e_{q} + {\text{i}}g_{q} \hfill \\ \end{gathered} \right.$$

The carbon emission-green power angle of nodes *p* and *q* can be expressed as:

9 $$\left\{ \begin{gathered} \angle C_{p} = \arctan \left( {\frac{{g_{p} }}{{e_{p} }}} \right) \hfill \\ \angle C_{q} = \arctan \left( {\frac{{g_{q} }}{{e_{q} }}} \right) \hfill \\ \end{gathered} \right.$$

If the carbon emission-green electricity amplitude angle of node, *p*, and the carbon emission-green electricity amplitude angle of node, *q*, are different and simplified, we can obtain:

10 $$\angle C_{p} - \angle C_{q} = \arctan \left( {\frac{{e_{q} g_{p} - e_{p} g_{q} }}{{e_{p} e_{q} + g_{p} g_{q} }}} \right)$$

It can be observed from the above equation that when the real part carbon intensity of nodes *p* and *q* is equal, i.e., $\angle C_{p} - \angle C_{q} = \arctan \left[ {e\left( {g_{p} - g_{q} } \right)/\left( {e_{p} e_{q} + g_{p} g_{q} } \right)} \right]$, the correlation between the imaginary part carbon potential, ${\text{g}}_{p}$ and ${\text{g}}_{q}$, of nodes *p* and *q* is proportional to the size, $\angle C_{p}$ and $\angle C_{q}$. When the carbon potential of the imaginary part of nodes *p* and *q* is equal, i.e., $\angle C_{p} - \angle C_{q} = \arctan \left[ {g\left( {e_{q} - e_{p} } \right)/\left( {e_{p} e_{q} + g_{p} g_{q} } \right)} \right]$, the size of the correlation, ${\text{e}}_{p}$ and ${\text{e}}_{q}$, is inversely proportional to $\angle C_{p}$ and $\angle C_{q}$. That is, when the real carbon intensity of the two nodes is equal, the carbon emission-green intensity angle, the proportion of new energy is larger, and the contribution rate of the carbon reduction is higher. When the imaginary carbon intensity of the two nodes is equal, the carbon emission-green electricity amplitude angle is larger, the physical carbon emission value is larger, and the carbon emission is lower. When the real and imaginary carbon intensity of the two nodes are not equal, the real carbon intensity is smaller and the carbon emission is lower.

## Application of complex structure carbon intensity

The carbon emission of the power generation side of the power system is equal to the carbon emission of the user’s electricity consumption along with the carbon emission of the network loss. In this section, the carbon responsibility allocation method of the user’s electricity consumption behavior and the carbon responsibility allocation method of the network loss are analyzed based on the complex structure carbon intensity theory.

### Carbon responsibility allocation method of user electricity behavior

According to the ‘source following load’ characteristic of the power system, the user side has the main share of the carbon responsibility, and the users’ electricity consumption behavior significantly affects the carbon intensity of each node. When there is a substantial increase in a user’s load demand corresponding to the day-ahead plan, part of the node carbon emission intensity becomes higher or the node carbon intensity is reduced. This produces constant power or power fluctuation of small load apportioned to more carbon emissions, and greater fluctuations in the load are instead apportioned to less carbon emissions, which violates the ‘who pollution, who governance, who pay’ the principle of fairness.

This study proposes a carbon responsibility allocation method for unit-load carbon flow correlation based on the theory of ‘complex structure carbon intensity’, as follows:

11 $$R_{{{\text{G, }}k}}^{{(E_{{{\text{G}},k}} \ne 0)}} = \alpha_{k} {\text{Re}} (R_{{\sum {\text{G}}}} )$$

12 $$R_{{{\text{G, }}k}}^{{(E_{{{\text{G}},k}} = 0)}} = \beta_{k} {\text{Im}} (R_{{\sum {\text{G}}}} )$$

13 $$\alpha_{k} = \frac{{{\text{Re}} ({\varvec{\eta}}_{{{\text{K, }}k}} {\varvec{R}}_{{\text{U - L}}} {\varvec{\zeta}}_{{\text{N}}}^{{\text{T}}} )}}{{{\text{Re}} ({\varvec{\zeta}}_{{\text{K}}} {\varvec{R}}_{{\text{U - L}}} {\varvec{\zeta}}_{{\text{N}}}^{{\text{T}}} )}}$$

14 $$\beta_{k} = \left\{ \begin{gathered} \frac{1}{{P_{{{\text{G}},\min }} }}\begin{array}{*{20}c} {} & {} & {} & {} & {} & {} & {P_{{{\text{G, }}k}}^{{(E_{{{\text{G}},k}} = 0)}} \le P_{{{\text{G}},\min }} } \\ \end{array} \hfill \\ \frac{{\frac{1}{{\left| {{\text{Im}} ({\varvec{\eta}}_{{{\text{K, }}k}} {\varvec{R}}_{{\text{U - L}}} {\varvec{\zeta}}_{{\text{N}}}^{{\text{T}}} )} \right|}}}}{{\sum {\frac{1}{{\left| {{\text{Im}} ({\varvec{\eta}}_{{{\text{K, }}k}} {\varvec{R}}_{{\text{U - L}}} {\varvec{\zeta}}_{{\text{N}}}^{{\text{T}}} )} \right|}}} }}\begin{array}{*{20}c} {} & {} & {P_{{{\text{G, }}k}}^{{(E_{{{\text{G}},k}} = 0)}} > P_{{{\text{G}},\min }} } \\ \end{array} \hfill \\ \end{gathered} \right.$$

Here, Re represents the real part of the complex structure; im denotes the imaginary part of the complex structure; ${\varvec{\eta}}_{{{\text{K}}, \, k}}$ indicates the K-dimensional row vector, where the *kth* element is 1 and the remaining elements are 0; $\alpha_{k}$ depicts the carbon responsibility sharing coefficient of the generator set whose physical carbon emission value is not zero; $\beta_{k}$ represents the carbon responsibility sharing coefficient of the generator set with a physical carbon emission value of zero; $P_{{{\text{G}},{\text{ min}}}}$ represents the minimum power generation threshold of the unit set when carbon responsibility is allocated.

Substitute (13) into (11):

15 $$R_{{{\text{G, }}k}}^{{(E_{{{\text{G}},k}} \ne 0)}} = {\text{Re}} \left( {\frac{{{\varvec{\eta}}_{{{\text{K, }}k}} {\varvec{R}}_{{\text{U - L}}} {\varvec{\zeta}}_{{\text{N}}}^{{\text{T}}} R_{{\sum {\text{G}}}} }}{{{\varvec{\zeta}}_{{\text{K}}} {\varvec{R}}_{{\text{U - L}}} {\varvec{\zeta}}_{{\text{N}}}^{{\text{T}}} }}} \right)$$

It can be observed from the above equation that, for the *kth* unit whose physical carbon emission value is not zero (not zero unit), the *kth* row of the unit-load carbon flow correlation matrix represents the contribution rate of unit, *k*, to the load, while ${\varvec{\eta}}_{{{\text{K, }}k}} {\varvec{R}}_{{\text{U - L}}} {\varvec{\zeta}}_{{\text{N}}}^{{\text{T}}}$ represents the total contribution rate. When ${\varvec{\eta}}_{{{\text{K, }}k}} {\varvec{R}}_{{\text{U - L}}} {\varvec{\zeta}}_{{\text{N}}}^{{\text{T}}}$ is larger, $R_{{{\text{G, }}k}}^{{(E_{{{\text{G}},k}} \ne 0)}}$ is larger, that is, more carbon emissions are apportioned by the *kth* generator unit with a physical carbon emission value of not zero.

The analysis of the *kth* generator set with zero physical carbon emission is similar. The difference is that $\alpha_{k}$ is positively correlated with the total contribution rate of unit, *k*, to load, while $\beta_{k}$ is inversely correlated. Therefore, for the generator set with zero physical carbon emission, when ${\varvec{\eta}}_{{{\text{K, }}k}} {\varvec{R}}_{{\text{U - L}}} {\varvec{\zeta}}_{{\text{N}}}^{{\text{T}}}$ is larger, $R_{{{\text{G, }}k}}^{{(E_{{{\text{G}},k}} \ne 0)}}$ is smaller. That is, the *kth* generator set with zero physical carbon emission has more carbon emissions. $\beta_{k}$ is inversely related to the total contribution rate of unit, *k*, to the load is inversely related. Therefore, when $P_{{{\text{G, }}k}}^{{(E_{{{\text{G}},k}} = 0)}} \le P_{{{\text{G}},\min }}$, that is, when the power generation is too small, it can result in various carbon emissions being allocated to the unit with insufficient power generation. Therefore, $\beta_{k}$ is set as a piecewise function to avoid several carbon emissions allocated to ensure the fairness principle of carbon responsibility allocation.

For the generation of units with non-zero physical carbon emission values, the greater the contribution to the nodes and loads, more carbon emissions are shared. For the generation of units with a physical carbon emission value of zero, the greater the contribution to the node and load, less carbon emissions are apportioned. The principle of fairness in the allocation of carbon responsibility is thus reflected, and its contribution to carbon reduction can be quantified based on the allocated carbon emissions.

A carbon responsibility allocation method for the users’ electricity consumption behavior is further developed based on the ‘complex structure carbon intensity’ theory. When a user’s load demand increases significantly corresponding to the day-ahead plan, the carbon flow calculation for the nodes passed by the flow circulation branch is performed by subtracting the load variation from the flow circulation branch flow. For the nodes with load changes, the node complex structure carbon potential correction is added as follows:

16 $$E_{{{\text{N, }}n}}^{{{\text{CN}}}} = \frac{{\sum\limits_{i = 1}^{N} {(P_{{{\text{B, }}in}} - \Delta P + \lambda P_{{{\text{Loss, }}in}} )E_{{{\text{N, }}in}} } + \sum\limits_{k = 1}^{K} {P_{{{\text{G, }}kn}} E_{{{\text{G, }}k}}^{{{\text{CN}}}} } }}{{\left( {\sum\limits_{i = 1}^{N} {P_{{{\text{B, }}in}} } + \sum\limits_{k = 1}^{K} {P_{{{\text{G, }}kn}} } } \right) - \Delta P}}$$

17 $$\Delta E_{{{\text{N, }}n}}^{{{\text{CN}}}} = \frac{{E_{{{\text{N, }}n}}^{{{\text{CN}}}} P_{{{\text{L, }}n}} + \Delta PE_{{\text{G, Slack}}}^{{{\text{CN}}}} }}{{P_{{{\text{L, }}n}} + \Delta P}} - E_{{{\text{N, }}n}}^{{{\text{CN}}}}$$

Here, $\Delta P$ represents the load variation, $\Delta E_{{{\text{N, }}n}}^{{{\text{CN}}}}$ represents the node complex structure carbon intensity correction, and $E_{{\text{G, Slack}}}^{{{\text{CN}}}}$ represents the unit complex structure carbon intensity of the balance node. The microscopic change process of each carbon index can be described in detail using Eq. (17). The specific flow of the carbon emissions can be accurately tracked using the carbon responsibility allocation method of users’ electricity consumption behavior, which presents effective tools to formulate the users’ carbon emission carbon tax policy.

### Network loss carbon responsibility allocation method

The power system network loss allocation generally allocates the network loss related costs to users receiving the network services according to the market rules. However, when calculating the carbon flow of the power system, the current research has transferred the carbon emission of the power generation side to the user side according to the theoretical analysis of the carbon emission flow. However, in an actual lossy network, a carbon responsibility allocation error will inevitably occur. Consequently, this section proposes a network loss carbon responsibility allocation method based on the ‘complex structure carbon intensity’.

In the conventional network loss carbon emission allocation method, the network loss carbon emission allocated by the user side is classified into the node carbon potential based on the network loss allocation coefficient, $\lambda$. The carbon emission allocated by the generation side can be expressed as:

18 $$R_{{\sum G }} = \zeta _{K} [diag(\user2{E}_{G} )\user2{P}_{G} ]\zeta _{N}^{T} - \zeta _{M} [\user2{P}_{L} diag(\user2{E}_{N} )]\zeta _{N}^{T}$$

Here, $R_{{\sum {\text{G}}}}$ represents the carbon emissions apportioned by the generation side;${\varvec{\zeta}}_{{\text{K}}}$ denotes the *K*-dimensional row vector, ${\varvec{\zeta}}_{{\text{N}}}$ refers to the *N*-dimensional row vector, where all the elements are 1; $\zeta_{{\text{K}}} [diag({\varvec{E}}_{{\text{G}}} ){\varvec{P}}_{{\text{G}}} ]\zeta_{{\text{N}}}^{{\text{T}}}$ represents the total carbon emissions generated by the generator set; and $\zeta_{{\text{M}}} [{\varvec{P}}_{{\text{L}}} diag({\varvec{E}}_{{\text{N}}} )]\zeta_{{\text{N}}}^{{\text{T}}}$ denotes the total carbon emissions shared by users.

After applying the complex structure carbon potential in the carbon flow theory, the unit + node carbon flow incidence matrix, ${\varvec{R}}_{{\text{U - N}}}$, considering network loss, can be expressed as:

19 $${\varvec{R}}_{{\text{U - N}}} = {\text{diag}}({\varvec{E}}_{{\text{G}}}^{{{\text{CN}}}} )\{ {\varvec{P}}_{{\text{N}}} [{\varvec{P}}_{{\text{N}}} - ({\varvec{P}}_{{\text{B}}} + \lambda {\varvec{P}}_{{{\text{Loss}}}} )^{{\text{T}}} ]^{ - 1} {\varvec{P}}_{{\text{G}}}^{{\text{T}}} \}^{{\text{T}}}$$

The unit + load carbon flow correlation matrix, ${\varvec{R}}_{{\text{U - L}}}$, is expressed as:

20 $${\varvec{R}}_{{\text{U - L}}} = {\varvec{R}}_{{\text{U - N}}} {\text{diag}}({\varvec{\zeta}}_{{\text{N}}} {\varvec{P}}_{{\text{L}}} ){\varvec{P}}_{{\text{N}}}^{{ - 1}}$$

The generation side and the load side are equally important to the power system in the power analysis. The generator set and load are interdependent in terms of energy and carbon emission responsibility sharing. The quantitative responsibility of the network loss carbon emission allocation on the power generation side and the user side can be calculated using Eqs. (19) and (20), and the fairness of the network loss carbon responsibility allocation can be realized.

The conventional carbon emission flow calculation method can be used to analyze the process and mechanism from the power generation side to the power consumption side, and to obtain the distribution of high-carbon nodes or branches in the network. The contribution of the carbon flow injection of all the generating units in the network to the node and load carbon flow rate can be obtained by using the unit-node carbon flow correlation matrix and the unit-load carbon flow correlation matrix. However, the *kth* row of the new energy unit, *k*, in the ${\varvec{R}}_{{\text{U - N}}}$ and ${\varvec{R}}_{{\text{U - L}}}$ matrices is an all-zero element. Therefore, it cannot reflect the contribution of the new energy unit injection to the node and load carbon reduction. Through the application of ‘complex structure carbon intensity’ in the carbon flow theory, ${\varvec{R}}_{{\text{U - N}}}$ and ${\varvec{R}}_{{\text{U - L}}}$ form a matrix composed of complex elements. This matrix can more clearly reflect the contribution of all the units to the nodes and loads without affecting the actual carbon emission calculation. It can also be used to determine the proportion of high green electricity. When compared to the node carbon potential matrix,${\varvec{E}}_{{\text{N}}}$, the node complex structure carbon intensity matrix,${\varvec{E}}_{{\text{N}}}^{{{\text{CN}}}}$, fully reflects the carbon reduction contribution of the new energy unit, and quantifies the performance. The higher the virtual carbon intensity, the greater is the contribution of the new energy unit in the node.

## Example analysis of test system

### 6-bus test system

**Figure 2:**
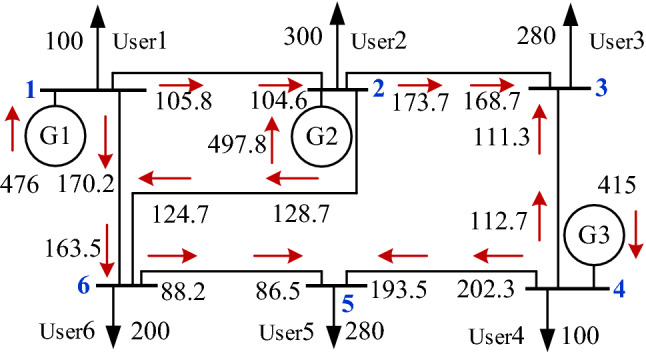
Steady-state power flow distribution of 6-bus test system.

The improved IEEE 6-bus test system was used for discussion and analysis. The topology of the 6-bus test system is shown in Fig. 2.

The generator parameters of the 6-bus test system are shown in Table 1.

**Table 1:** Parameters of the generator of the 6-bus test system.

No.	Generator Type	Capacity (MW)	Carbon complex intensity of Generator (tCO_2_/MWh)
G1	gas turbine unit	500	**0.3000** + 0.0000j
G2	renewable energy unit	500	0.0000 + **1.0000j**
G3	thermal power unit	500	**0.8750** + 0.0000j

Significance values are in bold.

The incidence matrix of the unit-load carbon emission flow of the 6-bus test system obtained by Eq. (20) is shown in Table 2.

**Table 2:** Unit-load carbon emission flow incidence matrix (complex structure carbon intensity).

No.	User 1	User 2	User 3	User 4	User 5	User 6
G1	60.0000	15.8068	9.1521	0	17.7015	40.1396
**G2**	**0**	**247.9084j**	**143.5389j**	**0**	**32.5479j**	**73.8048j**
G3	0	0	95.7950	85.0000	171.9550	0
Summation	60.0000	15.8068 + **247.9084j**	104.9471 + **143.5389j**	0	189.6565 + **32.5479j**	40.1396 + **73.8048j**

Significance values are in bold.

As shown in Table 2, after applying the complex structure carbon intensity, the unit-load carbon flow incidence matrix ${\varvec{R}}_{{\text{U - L}}}$ fully reflects the carbon reduction contribution of the new energy generator set G2 to different users (G2 row of the ${\varvec{R}}_{{\text{U - L}}}$ matrix).

The results were compared with the example in reference^11^. The comparison results show that the calculation results of the complex method of structure carbon intensity expression and carbon intensity expression method proposed here are exactly the same in the matrix row where the non-new energy unit is located, which demonstrates the correctness of the method proposed.

When $\lambda = 0.3$, that is, 30% of the network loss is allocated to the user side and 70% to the power generation side. The carbon emissions allocated by the generator set and the load are calculated according to Formulas (4) and (11)–(14), as shown in Table 3. The carbon flow rate in the table represents the carbon emissions allocated per unit of time.

**Table 3:** Carbon emission flow rate for generator and load.

No.	Unit contribution rate and load proportion	Carbon flow rate injected by the Unit (tCO_2_/h)	Carbon flow rate shared by unit and load (tCO_2_/h)
G1	34.2700%	142.8000	2.3918
G2	35.8400%	497.8000j	5.6357j
G3	29.8800%	352.7500	5.8979
Load 1	14.7100%	–	60.0000
Load 2	22.0600%	–	15.6812 + 247.9084j
Load 3	20.5900%	–	103.8585 + 140.6467j
Load 4	7.3500%	–	85.0000
Load 5	20.5900%	–	183.6964 + 31.4102j
Load 6	14.7100%	–	39.0242 + 72.1991j
Summation		495.5500 + 497.8000j	495.5500 + 497.8000j

According to Table 3, the greater the carbon flow rate injected into the unit with a nonzero physical carbon emission value, the greater the carbon emissions apportioned. The new energy unit is also allocated 5.6357j of carbon emissions, indicating the virtual carbon emissions allocated due to network losses.

Assuming that User 3 load increases from 280 to 400 MW compared to the day-ahead planned load, G1 is the equilibrium node in the system, and the complex structure carbon intensity changes in the 6-bus test system are shown in Table 4.

**Table 4:** Carbon intensity and complex structure carbon intensity in 6-bus test system.

No.	Before the surge		After the surge	
	Node complex structure carbon intensity (tCO_2_/MWh)	Real part of the load carbon flow rate (tCO_2_/h)	Node complex structure carbon intensity (tCO_2_/MWh)	Real part of the load carbon flow rate (tCO_2_/h)
Node 1	0.3000 + 0.0000j	60.0000	0.3000 + 0.0000j	60.0000
Node 2	**0.0527 + 0.8264j**	15.8068	**0.0938 + 0.6891j**	28.1312⬆
Node 3	**0.3748 + 0.5126j**	104.9471	**0.3083 + 0.5060j**	123.3355⬆
Node 4	0.8500 + 0.0000j	85.0000	0.8500 + 0.0000j	85.0000
Node 5	**0.6773 + 0.1162j**	189.6565	**0.6831 + 0.0969j**	191.2746⬆
Node 6	**0.2002 + 0.3690j**	40.1396	**0.2190 + 0.3077j**	43.8087⬆

Significance values are in bold.

According to Table 4, the complex structure carbon intensity of network nodes changes owing to the sudden increase in User 3 load, among which the complex structure carbon intensity of Node 1 remains unchanged. The real-part carbon intensity of Nodes 2, 5, and 6 increased, and the imaginary-part carbon intensity decreased. The carbon intensity of the complex structure of Node 3 decreased. The user loads at Nodes 2,5 and 6 do not change, and more carbon emissions are allocated because of the increase in the complex-structure carbon intensity, which violates the principle of fairness.

According to the carbon responsibility allocation method of users’ electricity consumption behavior described here, the complex structure carbon intensity and load carbon flow rate of each node calculated using Eqs. (16) and (17) are shown in Table 5.

**Table 5:** Carbon intensity and complex structure carbon intensity in 6-bus test system.

No.	Before the surge		After the surge	
	Node complex structure carbon intensity (tCO_2_/MWh)	Real part of the load carbon flow rate (tCO_2_/h)	Node complex structure carbon intensity (tCO_2_/MWh)	Real part of the load carbon flow rate (tCO_2_/h)
Node 1	0.3000 + 0.0000j	60.0000	0.3000 + 0.0000j	60.0000
Node 2	**0.0527 + 0.8264j**	15.8068	**0.0527 + 0.8264j**	15.8068
Node 3	**0.3748 + 0.5126j**	104.9471	**0.3524 + 0.5126j**	140.9471⬆
Node 4	0.8500 + 0.0000j	85.0000	0.8500 + 0.0000j	85.0000
Node 5	**0.6773 + 0.1162j**	189.6565	**0.6773 + 0.1162j**	189.6565
Node 6	**0.2002 + 0.3690j**	40.1396	**0.2002 + 0.3690j**	40.1396

Significance values are in bold.

The complex structure carbon intensity of Node 3 changed from 0.3748 + 0.5126j to 0.3524 + 0.5126j, and the complex structure carbon intensity of the other nodes remained unchanged. The carbon emissions of balancing unit G1 due to the sudden load increase of Node 3 are all attributed to the sudden load increase node, which ensures the fairness principle of carbon responsibility allocation.

### 30-bus test system

**Figure 3:**
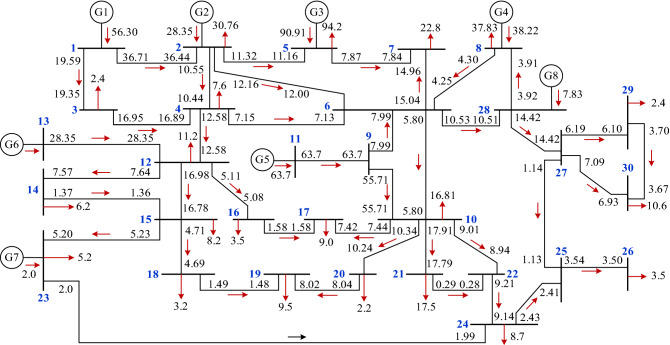
Steady-state power flow distribution of 30-bus test system.

This study presents a detailed discussion and analysis of the improved 30-bus test system to verify the effectiveness of carbon responsibility allocation and network loss carbon responsibility allocation methods. Figure 3 depicts the topology of the system.

Table 6 lists the generator parameters of the 30–node test system. Let $\lambda = 0.3$; the network loss is shared by the power generation side and the user side.

**Table 6:** Parameters of the generator of the 30-bus test system.

No.	Generator type	Capacity (MW)	Carbon complex potential of Generator (tCO_2_/MWh)
G1	Thermal power unit	60	**0.8750** + 0.0000j
G2	Wind turbine unit	30	0.0000 + **1.0000j**
G3	Hydropower unit	100	0.0000 + **1.0000j**
G4	Thermal power unit	40	**0.8750** + 0.0000j
G5	Gas turbine unit	70	**0.5250** + 0.0000j
G6	Photovoltaic unit	30	0.0000 + **1.0000j**
G7	Photovoltaic unit	10	0.0000 + **1.0000j**
G8	Wind turbine unit	10	0.0000 + **1.0000j**

Significance values are in bold.

When $\lambda = 0.3$, that is, when 30% of the network loss is allocated to the user side and 70% is allocated to the generation side, the load carbon flow rate and the unit injection carbon flow rate of the 30-bus test system can be obtained using Eqs. (4) and (5), as shown in Table 7.

**Table 7:** Load carbon emission flow rate and unit injected carbon emission flow rate.

Node	Load carbon emission rate (tCO_2_/h)	Unit injection carbon flow rate (tCO_2_/h)
1	0	**49.2625**
2	**15.1715** + 13.4596j	0 + 28.3500j
3	**2.1078**	0
4	**5.5658** + 1.2744j	0
5	**5.1018** + 88.4266j	0 + 90.9100j
6	0	0
7	**9.4458** + 10.5615j	0
8	**31.2421** + 1.9295j	**33.4425**
9	0	0
10	**8.9473** + 0.3378j	0
11	0	**33.4425**
12	**2.5210** + 8.3348j	0
13	0	0 + 28.3500j
14	**1.3994** + 4.6267j	0
15	**1.8525** + 6.1248j	0
16	**0.7892** + 2.6093j	0
17	**4.3089** + 1.3271j	0
18	**0.7239** + 2.3932j	0
19	**4.6199** + 1.2709j	0
20	**1.1744** + 0.0443j	0
21	**9.3334** + 0.3524j	0
22	0	0
23	**0.8499** + 4.2544j	0 + 2.0000j
24	**4.0775** + 1.4189j	0
25	0	0
26	**1.5114** + 1.0082j	0
27	0	0
28	0	0 + 7.8300j
29	**0.8321** + 1.3238j	0
30	**3.6844** + 5.8612j	0
Sum	**115.2601** + 156.9393j	**116.1475** + 157.4400j

Significance values are in bold.

Various expressions can be derived from the load carbon flow rate and the unit injection carbon flow rate presented in Table 7 by applying the complex structure carbon intensity. For instance, the load real carbon flow rate at Node 8 was as high as 31.2421, and the load real carbon flow rate at Node 18 was only 0.7239. The real load carbon flow rate at Nodes 4 and 5 did not vary significantly; however, the virtual load carbon flow rate at Nodes 4 and 5 varied significantly. In other words, the new energy consumption of Node 5 is much higher than that of Node 4.

Table 8 presents the node carbon intensity for the 30–node test system.

**Table 8:** The nodal complex carbon intensity of the 30–node test system.

Node	NCI (tCO_2_/MWh)	Node	NCI (tCO_2_/MWh)
1	**0.8750**	16	**0.2255** + 0.7455j
2	**0.4932** + 0.4376j	17	**0.4788** + 0.1475j
3	**0.8783**	18	**0.2262** + 0.7479j
4	**0.7323** + 0.1677j	19	**0.4863** + 0.1338j
5	**0.0542** + 0.9387j	20	**0.5338** + 0.0202j
6	**0.6020** + 0.2131j	21	**0.5333** + 0.0201j
7	**0.4143** + 0.4632j	22	**0.5337** + 0.0202j
8	**0.8259** + 0.0510j	23	**0.1634** + 0.8182j
9	**0.5250**	24	**0.4687** + 0.1631j
10	**0.5323** + 0.0201j	25	**0.4303** + 0.2871j
11	**0.5250**	26	**0.4318** + 0.2881j
12	**0.2251** + 0.7442j	27	**0.3452** + 0.5491j
13	0.0000 + 1.0000j	28	**0.3452** + 0.5491j
14	**0.2257** + 0.7462j	29	**0.3467** + 0.5516j
15	**0.2259** + 0.7469j	30	**0.3476** + 0.5529j

Significance values are in bold.

Combined with the analysis of Eq. (1), the node carbon intensity is only affected by the injection power flow, whereas Node 3 is only injected by Node 1. However, the carbon intensity of Node 3 is not equal to that of Node 1 because the network loss of Branch 1 → 2 is considered.

According to Table 8, Nodes 1, 3, 9, and 10 represent the real carbon intensity, Node 13 represents the imaginary carbon intensity, while the other nodes represent complex structure carbon intensity. From the 30-node topology presented in Fig. 2, Nodes 1, 3, 9, and 10 are only injected using thermal power units, Node 13 is only injected using the new G6 energy units, and the other nodes are the mixed power supply. The load at Nodes 3 and 29 is 2.4 MW. However, the carbon emissions shared vary owing to the two nodes’ different carbon intensities. Users can proceed with site selection from a carbon perspective. Conversely, the users at Node 3 can reduce the shared carbon emissions by building new energy units or actively purchasing new energy sources.

Table 9 presents the carbon emission apportionment of the generating units under three different distribution modes: apportionment by unit, generation ratio, and carbon flow correlation proposed here, which were obtained using Eqs. (6), (7), and (11)–(14).

**Table 9:** Carbon emission shared by generators under three different allocation methods.

No.	Unit Contribution rate	CEFR of generator (tCO_2_/h)		
		Case 1 (Unit share equally)	Case 2 (power generation proportion)	Case 3 (carbon flow correlation)
G1	17.84%	0.1109	0.1583	0.3743
G2	8.98%	0.1109	**0.0797**	0.1122j
G3	28.80%	0.1109	**0.2555**	**0.0348j**
G4	12.11%	0.1109	0.1075	0.2571
G5	20.18%	0.1109	0.1791	0.2561
G6	8.98%	0.1109	**0.0797**	0.1122j
G7	0.63%	0.1109	0.0056	0.1208j
G8	2.48%	0.1109	0.0220	0.1208j

Significance values are in bold.

Table 9 shows that the carbon emissions shared by each generator set cannot reflect fairness according to the method of unit sharing or power generation proportional sharing. For generating units with a nonzero physical carbon emission value, the greater the contribution to nodes and loads, the more carbon emissions are shared according to the proposed carbon flow-associated carbon responsibility allocation method. For generating units with a physical carbon emission value of zero, the greater the contribution to the node and load, the fewer carbon emissions are apportioned.

Furthermore, this example assumes that the generator set G5 in the 30-bus test system is a system balance-node unit. It is also assumed that the user connected to Node 24 exhibits a sudden increase in the load when compared to the day-ahead plan, from 8.7 to 20 MW. Based on the carbon responsibility allocation method of the proposed user’s electricity consumption behavior, the load carbon flow rates of Nodes 11 and 24 and the unit injection carbon flow rate are calculated, as shown in Table 10.

**Table 10:** Load carbon emission flow rate and unit injected carbon emission flow rate.

Node	Before user load change		After user load change	
	Load carbon emission rate (tCO_2_/h)	Unit injection carbon flow rate (tCO_2_/h)	Load carbon emission rate (tCO_2_/h)	Unit injection carbon flow rate (tCO_2_/h)
11	–	**33.4425**	**–**	**39.3750**
24	**4.0777** + 1.4190j	–	**10.0100** + 1.4190j	–

Significance values are in bold.

The complex carbon intensity of Node 24 changed from 0.4687 + 0.1631j to 0.5005 + 0.0568j, while the complex carbon intensity of the other nodes remained unchanged. As presented in Table 10, after the user load changes, the real part of the load carbon flow rate and the carbon flow rate injected into the system by the balancing unit increase, whereas the imaginary part remains unchanged. Compared to the carbon flow rate before the change, the difference between the carbon flow rate injected into the system by the balancing unit equals the difference between the carbon flow rate of the user load. In contrast, the complex carbon intensity of other nodes in the system remains unchanged. Therefore, the load carbon flow rates at the other nodes remained unchanged. In other words, the carbon emissions generated by the load surge of the balancing unit are all attributed to the load surge node, which ensures that the fairness principle of carbon responsibility sharing is satisfied.

According to the calculation results, the carbon intensity of the real part of the node increased when the node load suddenly increased. This indicates that the user shares more carbon emissions per unit of time and assumes more carbon responsibility. This method is crucial for users to consider day-ahead and intraday planning from a “carbon perspective.”

## Practical application of complex structure carbon emission flow theory

The analysis presented above demonstrates that in the conventional carbon emission flow calculation method, the *kth* row of the new energy unit, *k*, in the ${\varvec{R}}_{{\text{U - N}}}$, and ${\varvec{R}}_{{\text{U - L}}}$ matrices contains only zero elements. Following the application of the “complex structure carbon intensity” in the carbon emission flow theory, ${\varvec{R}}_{{\text{U - N}}}$ and ${\varvec{R}}_{{\text{U - L}}}$ form a matrix composed of complex elements. The *kth* row of the new energy unit, *k*, in the matrix becomes the carbon flow element, reflecting the contribution of node green electricity. Therefore, this study proposes a carbon responsibility allocation method based on the complex structure carbon emission flow theory, which solves the problems of unequal carbon responsibility allocation of new energy units and unequal proportion of green power at the same carbon intensity nodes.

**Figure 4:**
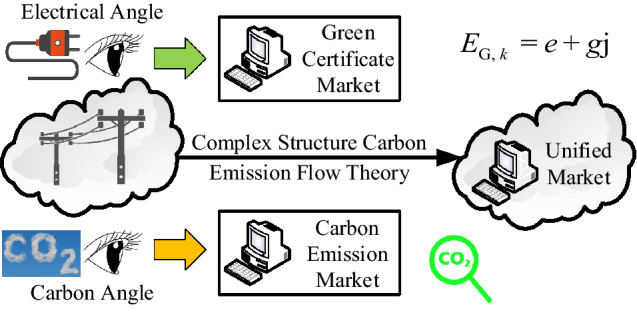
Practical application of complex structure carbon emission flow theory.

Furthermore, China has not yet developed efficient green certificate or carbon emission markets. The existing green certificate system has not performed well in promoting renewable energy integration or alleviating the pressure on financial subsidies. Meanwhile, the existing carbon market has not performed well in promoting emission reduction^22^. The interaction between existing carbon trading and green power trading can better reflect the low-carbon nature of new energy and help improve the implementation mechanism of clean alternatives to integrated energy systems^23^. China has also played a leading role in successfully formulating policies regarding the lowest proportion of green electricity in electricity consumption, promoting industry-leading enterprises, large state-owned enterprises, multinational companies, and other enterprises to consume green electricity. Regions with more export-oriented enterprises and strong economic affordability have been promoted to increase the proportion of green electricity consumption gradually. Therefore, two major constraints are expected in the future: carbon emission constraints and the proportion of green electricity constraints. The means of providing solutions for low-carbon development based on carbon emission and green electricity constraints must be considered. The “complex structure carbon intensity” proposed here realizes the joint analysis of the carbon emission and green electricity proportion of user nodes by tracking the “carbon trajectory” and “green electricity trajectory” in the system. This can provide a solution for future power optimization applications that will face carbon emissions and green electricity proportion constraints. The practical application of the complex-structure carbon emission flow theory is shown in Fig. 4.

## Conclusion

This study analyzed the quantitative allocation of power carbon responsibility driven by dual carbon targets from the “carbon perspective”. It introduced the concept of “complex structure carbon intensity” and defined the “carbon emission-green electricity amplitude angle” based on the carbon emission flow theory. The proportion of green electricity (green electricity trajectory) can also be calculated because the carbon emission flow (carbon trajectory) calculation was not affected. Subsequently, this study proposed a carbon responsibility allocation method for network loss and the carbon responsibility allocation method of user electricity consumption behavior based on the application of complex structure carbon intensity in the carbon emission flow theory. Applying the proposed complex structure carbon intensity in the carbon emission flow theory expands the research dimensions of power carbon emissions for low-carbon development from the “carbon perspective.” This presents a new optimization space for the operation of distribution networks, and the carbon emission flow theory becomes a bridge between calculation evaluations and optimization decisions.

The main conclusions and innovations of this study are summarized as follows:

1. The application of complex structure carbon intensity in carbon emission flow theory helps quantify new energy’s contribution to carbon reduction. It also solves the problem of ambiguity in the contribution of new energy units to nodes and loads. Furthermore, it expands the research dimensions of power carbon emissions and presents a new optimization space for the low-carbon operation of the distribution network. Consequently, the carbon flow theory acts as a bridge between calculation, evaluation, and optimization decision making.
2. By applying the “complex structure carbon intensity” in the carbon flow theory, the *kth* row of the new energy unit, *k*, in the ${\varvec{R}}_{{\text{U - N}}}$ and ${\varvec{R}}_{{\text{U - L}}}$ matrices contains only zero elements and becomes a complex structural element. The contribution of all units to the nodes and loads can be more clearly reflected without affecting the actual carbon emission calculation, and the nodes with a high green power ratio can be determined.
3. Based on the complex structure carbon intensity theory and the carbon emission-green electric amplitude angle, when the real carbon intensities of the two nodes are equal, the carbon emission-green electric amplitude angle is larger, the proportion of new energy is larger, and the contribution rate of carbon reduction is higher. When the imaginary carbon potentials of the two nodes are equal, the carbon emission-green electricity amplitude angle is larger, the physical carbon emission value is larger, and the carbon emission is lower. When the two nodes’ real and imaginary carbon intensities are unequal, the real carbon intensity is smaller, and the carbon emissions are lower.
4. Based on the carbon responsibility allocation method of users’ electricity consumption behavior and carbon responsibility allocation method of the network loss described here, the fairness principle of carbon responsibility allocation is ensured. Furthermore, the reverse driving force of user participation in carbon emission reduction was improved. Therefore, power plants and users can make next-stage optimization decisions based on allocated carbon emissions.
5. The “complex structure carbon intensity” proposed here realizes the joint analysis of the carbon emission and green electricity proportion of user nodes by tracking the “carbon trajectory” and “green electricity trajectory” in the system. This can provide a solution for future power optimization applications that will face carbon emissions and green electricity proportion constraints.

Future work will focus on analyzing the low carbonization of electricity driven by the dual carbon goal from the “carbon perspective.” This study aims to improve the carbon emission flow theory by applying the complex structure carbon intensity in the carbon emission flow theory. It also aims to promote low-carbon development of electricity in the future.

## Data Availability

All data generated or analyzed during this study are included in this published article.
